# MAP3K1 function is essential for cytoarchitecture of the mouse organ of Corti and survival of auditory hair cells

**DOI:** 10.1242/dmm.023077

**Published:** 2015-12-01

**Authors:** Rizwan Yousaf, Qinghang Meng, Robert B. Hufnagel, Ying Xia, Chandrakala Puligilla, Zubair M. Ahmed, Saima Riazuddin

**Affiliations:** 1 Department of Otorhinolaryngology Head & Neck Surgery, School of Medicine, University of Maryland, Baltimore, MD 21201, USA; 2 Department of Environmental Health, University of Cincinnati, College of Medicine, Cincinnati, OH 45267, USA; 3 Divisions of Pediatric Ophthalmology and Human Genetics, Cincinnati Children's Hospital Medical Center, University of Cincinnati, Cincinnati, OH 45229, USA; 4 Department of Pathology & Laboratory Medicine, Medical University of South Carolina, Charleston, SC 29425, USA

**Keywords:** *Map3k1*, *Mekk1*, *Fgfr3*, *Fgf8*, *Fgf10*, Hearing loss, Supernumerary outer hair cells, MAPK pathway, FGF signaling pathway

## Abstract

MAP3K1 is a serine/threonine kinase that is activated by a diverse set of stimuli and exerts its effect through various downstream effecter molecules, including JNK, ERK1/2 and p38. In humans, mutant alleles of *MAP3K1* are associated with 46,XY sex reversal. Until recently, the only phenotype observed in *Map3k1^tm1Yxia^* mutant mice was open eyelids at birth. Here, we report that homozygous *Map3k1^tm1Yxia^* mice have early-onset profound hearing loss accompanied by the progressive degeneration of cochlear outer hair cells. In the mouse inner ear, MAP3K1 has punctate localization at the apical surface of the supporting cells in close proximity to basal bodies. Although the cytoarchitecture, neuronal wiring and synaptic junctions in the organ of Corti are grossly preserved, *Map3k1^tm1Yxia^* mutant mice have supernumerary functional outer hair cells (OHCs) and Deiters' cells. Loss of MAP3K1 function resulted in the downregulation of *Fgfr3*, *Fgf8*, *Fgf10* and *Atf3* expression in the inner ear. Fgfr3, Fgf8 and Fgf10 have a role in induction of the otic placode or in otic epithelium development in mice, and their functional deficits cause defects in cochlear morphogenesis and hearing loss. Our studies suggest that MAP3K1 has an essential role in the regulation of these key cochlear morphogenesis genes. Collectively, our data highlight the crucial role of MAP3K1 in the development and function of the mouse inner ear and hearing.

## Abstract (precis)

**Summary:** *Map3k1* mutant mice exhibit early-onset profound hearing loss and supernumerary outer hair cells, along with dysregulation of the FGF signaling pathway, accentuating its function in otic epithelium development and morphogenesis.

## INTRODUCTION

Mitogen-activated protein kinases (MAPKs) are responsible for regulating a wide array of cellular functions and processes. The MAPK signaling cascade consists of three tiered phosphorylation steps, starting with the phosphorylation of MAPK kinase kinases (MAP3Ks, MEK kinases or MKKKs) in response to a plethora of stimuli, which in turn phosphorylate the MAPK kinases (MAP2Ks, MEK or MKKs) and then the MAPKs (Kyriakis and Avruch, 2001; Uhlik et al., 2004). MAP3K1, a member of the MAPK kinase kinases family, plays a diverse cell-signaling function in various biological systems, including immune system development and function (Gallagher et al., 2007; Labuda et al., 2006), vasculature remodeling (Li et al., 2005), tumor progression (Cuevas et al., 2006), cardiogenesis (Minamino et al., 2002), and injury repair (Deng et al., 2006).

MAP3K1 belongs to the serine/threonine kinase class that also participates in the regulation of the MAPK cascade (Cuevas et al., 2007; Hagemann and Blank, 2001; Uhlik et al., 2004). The MAP3K1 protein contains an ubiquitin interaction motif (UIM), a caspase-3 cleavage site and a conserved kinase domain (Uhlik et al., 2004; Witowsky and Johnson, 2003). MAP3K1 is associated with the plasma membrane and is tethered by α-actinin to actin stress fibers and by protein tyrosine kinase 2 (PTK2) to focal adhesions in cells (Christerson et al., 2002; Cuevas et al., 2003). Following an apoptotic signal, caspase-3 cleaves MAP3K1 at phosphorylated Asp874 (p.Asp874), resulting in the separation of the N-terminal UIM motif from the 91-kDa C-terminal kinase domain, releasing it from the cell membrane into the cytosol (Bonvin et al., 2002; Schlesinger et al., 2002). MAP3K1 is activated in response to a number of different stimuli, such as cold, growth factors, mild hyperosmolarity, microtubule disruption, cell shape disturbance, pro-inflammatory cytokines and other physiological stresses (Sadoshima et al., 2002; Xia et al., 2000; Yujiri et al., 1998). Once activated, MAP3K1 exerts its effect through the JNK, ERK1/2 and p38 MAPK pathways, as well as the transcription factors Jun and NF-κB (Bonvin et al., 2002; Guan et al., 1998; Xia et al., 2000; Yujiri et al., 1998).

In humans, mutant alleles of *MAP3K1* are associated with 46,XY gonadal dysgenesis (Pearlman et al., 2010). These gain-of-function alleles affect the downstream phosphorylation of p38 and ERK1/2, as well as binding of MAP3K1 with the cofactors RHOA and MAP3K4 (Loke and Ostrer, 2012). Additionally, *in vitro* studies have suggested that these mutations alter the sex-determination pathway by concomitantly upregulating β-catenin expression and downregulating expression of the *SOX9*, *SRY*, *FGF9* and *FGFR2* genes (Loke et al., 2014). However, mice carrying the *Map3k1* loss-of-function allele display a minor testicular deficit in the developing gonad and have normal gross appearance besides the open-eyelid phenotype (Warr et al., 2011). MAP3K1-deficient mice display an eye open at birth (EOB) phenotype (Zhang et al., 2003) and have immune-system and wound-healing deficits, abnormal retinal vascularization, disintegration of retinal pigment epithelium, loss of photoreceptors, and retinal degeneration (Mongan et al., 2011). Additionally, cultured keratinocytes from these mutant mice display a lack of actin stress fiber formation and deficient cell migration (Yujiri et al., 2000; Zhang et al., 2003).

Previous studies have demonstrated the role of MAPK-mediated fibroblast growth factor (FGF) signaling in otic induction and development (Urness et al., 2010). Hearing depends on the precise organization of sensory hair cells and non-sensory supporting cells within the organ of Corti (OC). Any substantial alteration in the cellular number, alignment or patterning within the OC causes hearing loss, underlining the necessity of defined cytoarchitecture in the OC for sound perception. Here, we report that mice lacking the kinase domain of MAP3K1 (*Map3k1^tm1Yxia^*) have an extra row of OHCs and supporting cells in the OC and suffer from early-onset profound hearing loss. Our results demonstrate the indispensable role of MAP3K1 in mouse inner-ear development and function.

**TRANSLATIONAL IMPACT**

**Clinical issue**

Hearing loss, which can present during early life or as a late-onset condition, is one of the most common neurosensory disorders worldwide. Normal hearing depends on the precise organization of sensory hair cells and non-sensory supporting cells within the organ of Corti (OC) in the cochlea of the ear. Any significant alterations to cell number, alignment or patterning within the OC causes hearing loss, underlining the importance of a defined cytoarchitecture in the OC for sound perception. Thus, understanding the molecular signaling cascades that lead to inner-ear sensory-cell differentiation and function is important for defining the molecular basis of hearing loss and devising strategies for hearing restoration. In this study, the putative role of MAP3K1 (a serine/threonine kinase with a pivotal function in MAPK signal transduction cascades) in inner-ear development and function was explored in mice.

**Results**

To determine whether MAP3K1 plays a part in hearing, the authors characterized homozygous *Map3k1^tm1Yxia^* kinase-null mutant mice. In the mouse inner ear, MAP3K1 is localized at the apical surface of cochlear supporting cells, in close proximity to basal bodies. A hearing test of the mutant mice revealed early-onset profound hearing loss, along with progressive degeneration of outer hair cells (OHCs). The authors show that the cytoarchitecture, neuronal wiring and synaptic junctions in the OC are unaffected; however, loss of MAP3K1 function results in an extra row of functional OHCs and Deiters’ cells (a type of cochlear supporting cell). Loss of MAP3K1 function also results in downregulation of members of the fibroblast growth factor (FGF) signaling pathway: Fgfr3, Fgf8, Fgf10 and Atf3 expression in the inner ear. Previous studies have shown that Fgfr3, Fgf8 and Fgf10 have a role in induction of the otic placode – from which the auditory system develops during embryogenesis – or in otic epithelium development in mice.

**Implications and future directions**

Functional deficits in the FGF signaling pathway are known to cause defects in cochlear morphogenesis and hearing loss in mice. This study provides evidence that MAP3K1 has an essential role in the regulation of the FGF signaling pathway during the development, function and survival of inner-ear hair cells. Homozygous *Map3k1^tm1Yxia^* mice represent another model for the investigation of signaling pathways involved in hearing loss. In addition, elucidation of the central role of the MAP3K1 kinase protein could have implications for the controlled regeneration of inner-ear sensory cells for hearing restoration.

## RESULTS

### MAP3K1 is localized with basal bodies in supporting cells

**Fig. 1.**
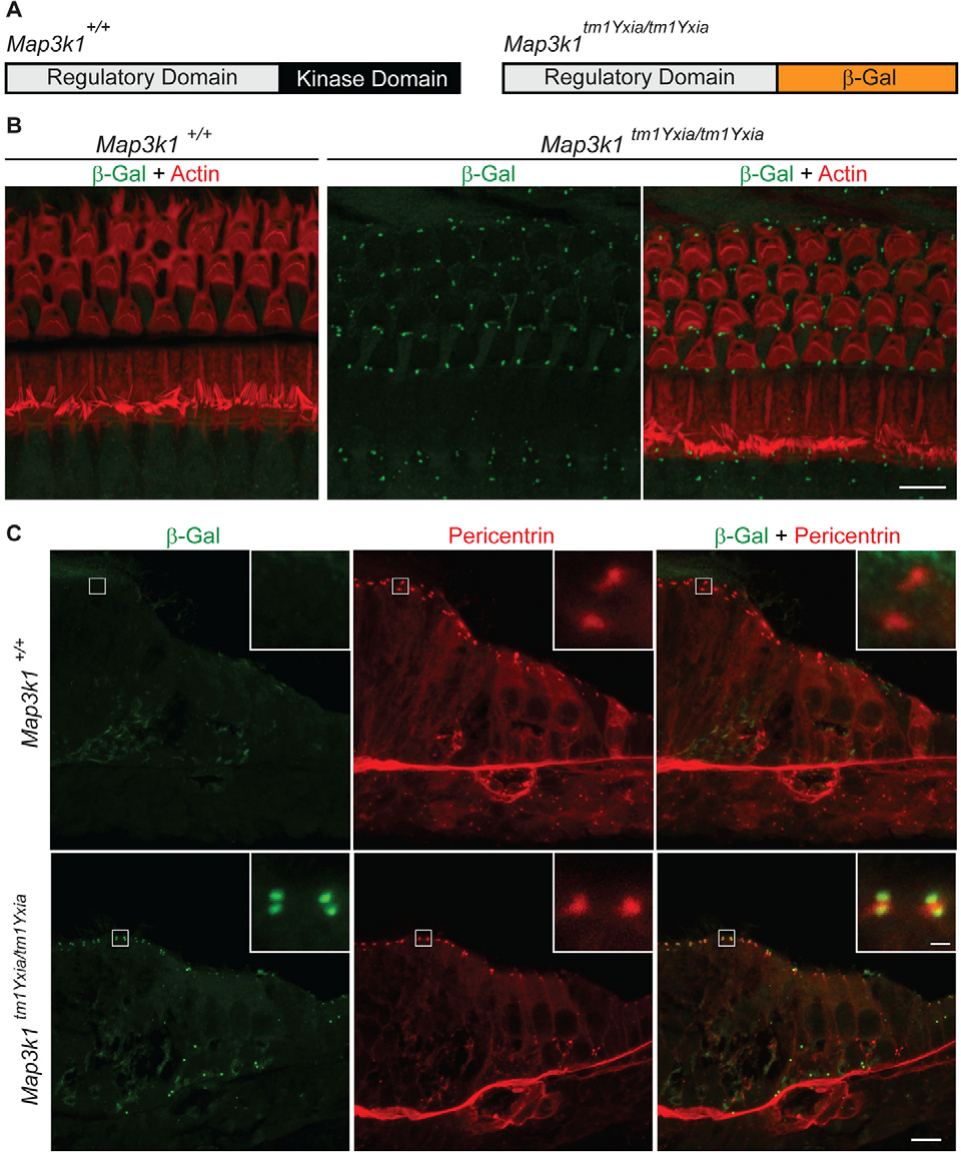
**Localization of MAP3K1 in the inner ear.** (A) MAP3K1 has two domains, a regulatory domain and a kinase domain, whereas, in *Map3k1* mutant mice, the kinase domain has been replaced with a β-galactosidase reporter cassette, resulting in a MAP3K1–β-galactosidase fusion protein. (B) Confocal imaging of the whole-mount preparation of organ of Corti (OC) from P16 mice labeled with the anti-β-galactosidase antibody (green) and phalloidin (red). As anticipated, no β-galactosidase labeling was observed in control (*Map3k1^+/+^*) mice, whereas, in the *Map3k1^tm1Yxia^* mutant mice, a distinct expression of the MAP3K1–β-galactosidase fusion protein was observed at the apical surface of supporting cells of the OC. Scale bar: 10 μm. (C) In sections of P0 mice, the MAP3K1–β-galactosidase fusion protein (green) colocalizes with pericentrin (red), a marker for basal cell bodies. In addition, weak diffused cytoplasmic expression of fusion protein was also observed in inner and outer hair cells. Scale bar: 10 μm. Insets show higher magnification of β-galactosidase and pericentrin localization. Scale bar: 1 μm.

To comprehend the role of *Map3k1* in inner-ear development and function, we used previously generated *Map3k1^tm1Yxia^* mice (Xia et al., 2000; Zhang et al., 2003). In these mice, the exons encoding the kinase domain of MAP3K1 have been replaced with the bacterial *lacZ* gene, resulting in the expression of a MAP3K1–β-galactosidase fusion protein (Fig. 1A). Immunolabeling of the whole-mount preparation for the OC from these mice revealed punctate expression of the MAP3K1–β-galactosidase fusion protein at the apical surface of supporting cells (Fig. 1B). Interestingly, the MAP3K1–β-galactosidase fusion protein and basal-body marker pericentrin colocalize with a very exquisite pattern (Fig. 1C). The MAP3K1–β-galactosidase fusion protein was expressed as two puncta on either side of the pericentrin-labeled basal cell bodies (Fig. 1C, inset). Basal bodies are known to direct planar cell polarity (PCP) of sensory hair cells (Jones et al., 2008). Therefore, we investigated the orientation of the OHC bundles in *Map3k1* mutant mice, and no significant deficit was observed (see Fig. S1).

Additionally, weak diffused cytoplasmic expression of *Map3k1* was also observed in the inner hair cells and outer hair cells (Fig. 1C), and in the marginal and intermediate cells of the stria vascularis (see Fig. S2A). Moreover, β-galactosidase staining in adult *Map3k1* heterozygous mice revealed expression in supporting cells of the lesser epithelial ridge, greater epithelial ridge, stria vascularis, Reissner's membrane and the spiral ganglion neurons (see Fig. S2B).

### *Map3k1^tm1Yxia/tm1Yxia^* mice have supernumerary outer hair cells and Deiters’ cells

**Fig. 2.**
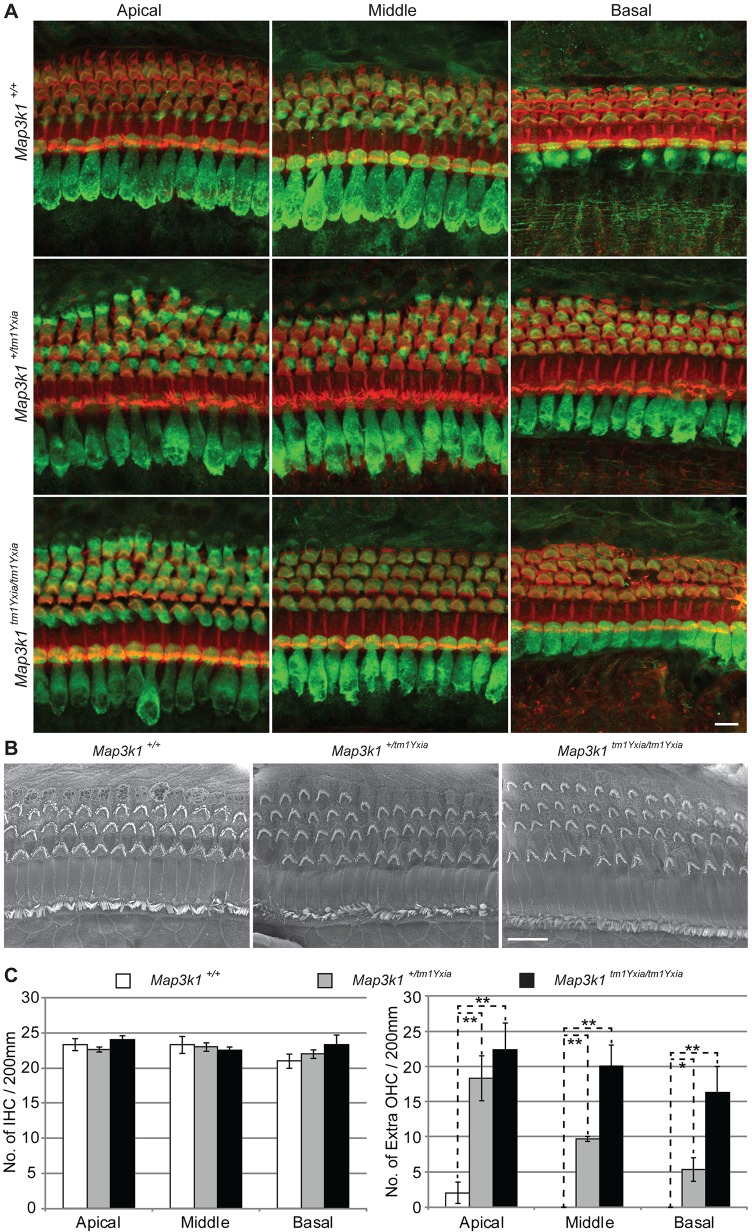
***Map3k1^tm1Yxia^* mutant mice have supernumerary outer hair cells (OHCs).** (A) Whole-mount preparation of the OC from P12 mice labeled with myosin VIIa (green) and phalloidin (red). In contrast to three rows of OHCs observed in wild-type mice, *Map3k1^tm1Yxia^* mutant mice have an extra row of OHCs along the length of the cochlea, whereas sparse patches of a fourth row of OHCs was observed in *Map3k1^tm1Yxia^* heterozygous mice. Scale bar: 10 μm. (B) Scanning electron micrographs of P14 mice revealed characteristic polarized ‘V’-shaped stereocilia bundles at the apical surface of supernumerary OHCs present in *Map3k1^tm1Yxia^* heterozygous and homozygous mutant mice. Scale bar: 10 μm. (C) Quantitation of inner hair cells (IHCs) and OHCs in control and *Map3k1^tm1Yxia^* mice at P12. For quantification purposes, organ of Corti (OC) were isolated from four wild-type and *Map3k1^tm1Yxia^* mutant mice each and hair cells were counted in the apical middle and basal coil regions. No significant difference was observed in the IHC number. Statistically significant (*P<0.05, **P<0.01) increases in the OHC number were observed in the *Map3k1^tm1Yxia^* homozygous mutant and heterozygous mice, with a gradient from apex to base (mean±s.e.m.).

Upon confocal imaging of the OC from *Map3k1^tm1Yxia^* heterozygous and homozygous mice, we observed supernumerary outer hair cells (OHCs) throughout development (Fig. 2). The *Map3k1^tm1Yxia^* heterozygous mice have sparse one- to ten-cell stretches of an extra row of OHCs, whereas homozygous *Map3k1^tm1Yxia^* mice have a continuous extra row of OHCs in the apical, middle and basal cochlear turns (Fig. 2A). These supernumerary OHCs also have correctly polarized mechanosensitive hair bundles at their apical poles (Fig. 2B). Although no difference was observed in the number of inner hair cells (IHCs) (Fig. 2C), a statistically significant increase in OHCs was observed throughout the cochlear duct in homozygous and heterozygous *Map3k1^tm1Yxia^* mice (Fig. 2C). However, in comparison with homozygous mutant mice, fewer extra OHCs were observed throughout the cochlear duct in heterozygous *Map3k1^tm1Yxia^* mice (Fig. 2C), suggesting a dose-dependent role of MAP3K1-mediated signaling in the cytoarchitecture of the mouse OC.

**Fig. 3.**
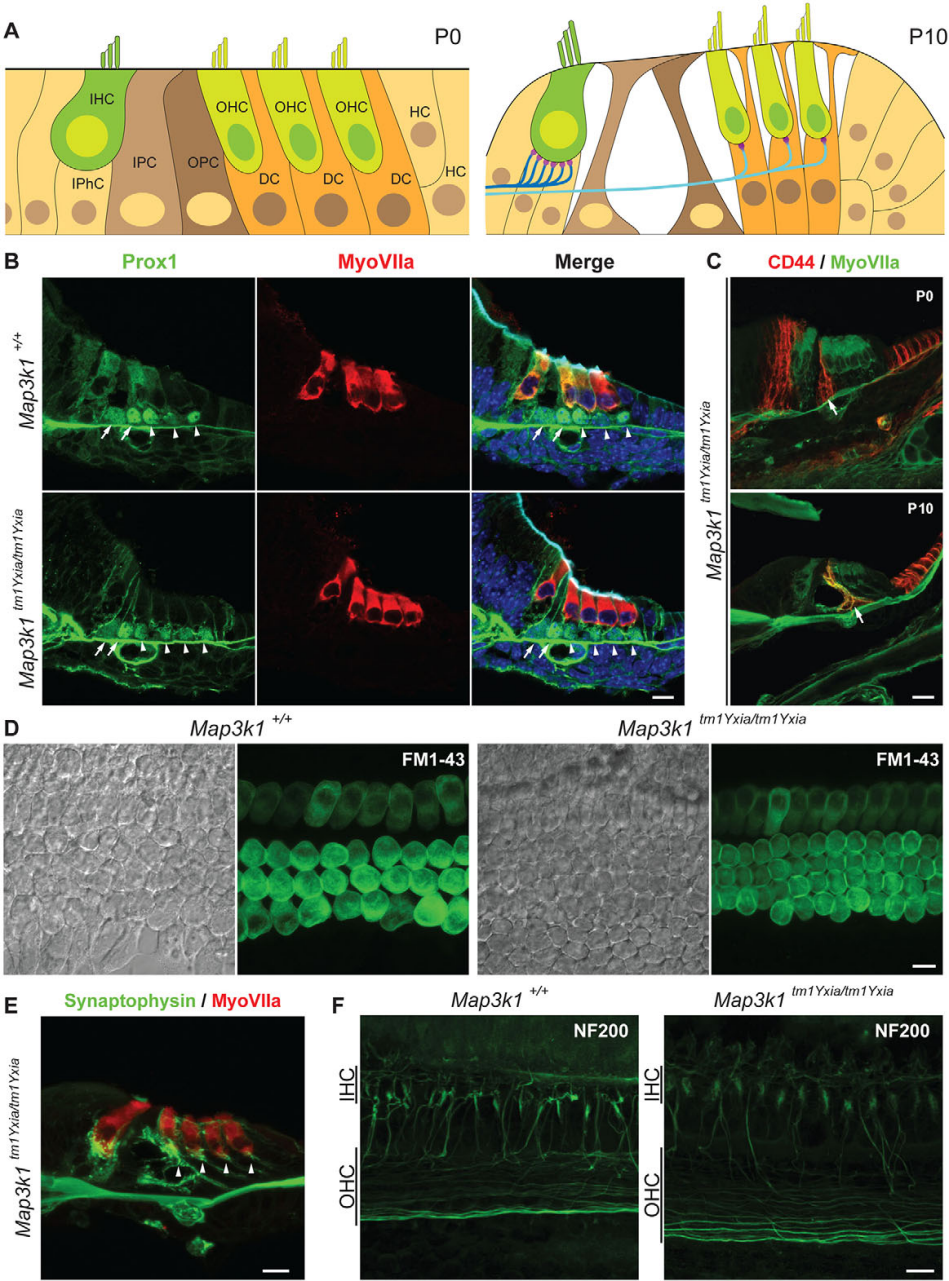
**Cytoarchitecture of the organ of Corti (OC) is preserved in *Map3k1^tm1Yxia^* mutant mice.** (A) Schematic representation of the developing mouse OC at P0 and P10. OHC, outer hair cell; IHC, inner hair cell; DC, Deiters' cell; OPC, outer pillar cell; IPC, inner pillar cell; HC, Hensen's cells; IPhC, inner phalangeal supporting cells. (B) Cross-section of *Map3k1^tm1Yxia^* mutant and wild-type control mice at P0, immunostained with Prox1 (green) and myosin VIIa (red). The arrows point to the pillar cells, whereas Deiters' cells are marked by the arrowheads. *Map3k1^tm1Yxia^* mutant mice have an extra row of OHCs accompanied by an extra row of Deiters' cells. Scale bar: 10 μm. (C) Immunostaining with the anti-CD44 antibody, a marker for OPCs, including Claudius cells, revealed an intact gross cytoarchitecture of the OC in *Map3k1^tm1Yxia^* mutant mice at P0 and P10. Scale bar: 10 μm. (D) No apparent difference in the FM1-43 dye uptake was observed among control and *Map3k1^tm1Yxia^* mutant explants. FM1-43 dye was also taken up by the supernumerary OHCs present in *Map3k1^tm1Yxia^* mutant mice. Scale bar: 10 μm. (E) Supernumerary OHCs in *Map3k1^tm1Yxia^* mutant mice are innervated and have synaptic junctions (arrowheads), immunolabeled with synaptophysin (green). Scale bar: 10 μm. (F) *Map3k1^tm1Yxia^* mutant mice have grossly intact neuronal wiring. Neurofilament (NF-200) protein immunostaining of wild-type control and *Map3k1^tm1Yxia^* mutant mice revealed grossly intact neuronal wiring of an extra row of OHCs. Scale bar: 10 μm.

In higher vertebrates, each hair cell in the OC is enveloped by supporting cells (Fig. 3A). In the mouse OC, the IHCs rest on the inner phalangeal cells, whereas each OHC rests upon a single Deiters’ cell, and inner and outer pillar cells separate these two types of sensory hair cells (Fig. 3A). To determine the effect of extra rows of OHCs on the precise cytoarchitecture of the OC, we immunolabeled the cochlear sections from postnal day 0 (P0) control and *Map3k1^tm1Yxia^* mutant mice with an anti-Prox1 antibody, a marker for pillar and Deiters' supporting cells. In *Map3k1^tm1Yxia^* mutant mice, the extra row of OHCs was also supported by an extra row of Deiters' cells (Fig. 3B). However, the organization of the inner and outer pillar cells, Claudius cells and the tunnel of Corti was not affected in *Map3k1^tm1Yxia^* mutant mice (Fig. 3B,C). These findings suggest that MAP3K1 has a role in the development of controlled cytoarchitecture of mouse OC.

To assess the functional status of supernumerary hair cells, we briefly exposed (15 s) the OC explants from control and *Map3k1^tm1Yxia^* mutant mice to FM1-43, a styryl pyridinium dye that enters the hair cells via partially open mechanotransduction channels at rest (Gale et al., 2001; Meyers et al., 2003). Hair cells from control and *Map3k1^tm1Yxia^* mice showed dye loading in all rows of sensory cells, including the extra row of OHCs (Fig. 3D). We also performed synaptophysin immunostaining to identify postsynaptic endings of efferent neurons forming axodendritic synapses with the dendrites of spiral ganglion neurons in P12 control and mutant mice (Fig. 3E). Interestingly, the synaptophysin labeling was also observed at the base of an extra row of OHCs (Fig. 3E, arrowhead). Furthermore, no obvious difference in the neuronal wiring of the OC in the control and *Map3k1^tm1Yxia^* mutant mice was observed at P12 (Fig. 3F). However, the width of the area of neurons around the OHCs was wider in *Map3k1^tm1Yxia^* mutant mice, likely due to the wiring of the extra row of OHCs (Fig. 3F). Collectively, we observed that *Map3k1^tmYxia^* mutant mice have a completely developed, structurally supported, polarized and functional extra row of innervated OHCs and Deiters' cells.

### *Map3k1^tm1Yxia^* mutant mice have early-onset hearing loss

**Fig. 4.**
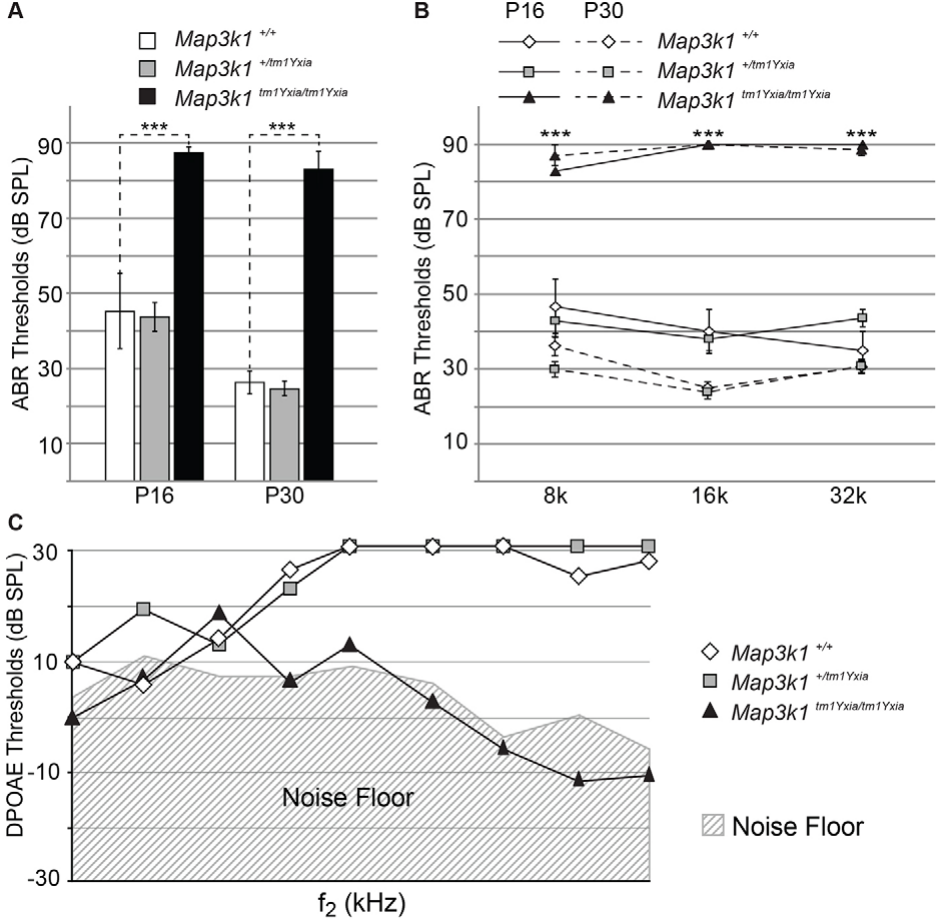
***Map3k1^tm1Yxia^* mutant mice have elevated hearing thresholds.** Hearing thresholds of 3 wild-type, 7 heterozygous and 8 *Map3k1^tm1Yxia^* homozygous mice were evaluated at P16, whereas 8 wild-type, 11 heterozygous and 7 *Map3k1^tm1Yxia^* homozygous mice were tested at P30. (A) Averaged auditory brainstem responses (ABR) thresholds of wild-type, and heterozygous and homozygous *Map3k1^tm1Yxia^* mice at P16 and P30 in response to click stimulus. The *Map3k1^tm1Yxia^* mutant mice showed significantly (***P<0.001) elevated thresholds compared with heterozygous and wild-type mice at both ages (mean±s.e.m.). (B) Averaged ABR thresholds of wild-type (white diamonds), heterozygous (gray squares) and homozygous *Map3k1^tm1Yxia^* mutant (black triangles) mice at P16 (solid lines) and P30 (dashed lines), in response to 8-kHz, 16-kHz and 32-kHz tone-bursts. At both developmental stages, *Map3k1^tm1Yxia^* mutant mice showed significantly (***P<0.001) elevated thresholds compared with the wild-type control and heterozygous mice at all frequencies tested (mean±s.e.m.). (C) Distortion product otoacoustic emissions (DPOAEs) of *Map3k1^tm1Yxia^* mutant (black triangles), heterozygous (gray squares) and wild-type control (white diamonds) mice at P30, represented as a function of f2 stimulus frequencies. *Map3k1^tm1Yxia^* mutant mice showed no responses, with values close to the noise floor, indicating that the residual OHCs were non-functional. SPL, sound pressure level.

Next, to assess the hearing function of *Map3k1^tm1Yxia^* mutant mice, we measured auditory brainstem responses (ABRs) and distortion product otoacoustic emissions (DPOAEs). Although the wild-type control and *Map3k1^tm1Yxia^* heterozygous mice had comparable hearing thresholds across all the tested frequencies, significant hearing loss was observed in the homozygous *Map3k1^tm1Yxia^* mice at P16 and P30 (Fig. 4A,B). Similarly, the DPOAEs of wild-type and *Map3k1^tm1Yxia^* heterozygous mice were comparable, whereas the homozygous *Map3k1^tm1Yxia^* mice had no detectable DPOAE thresholds (Fig. 4C). Taken together with ABRs, these results suggest that hearing loss in *Map3k1^tm1Yxia^* mutant mice is likely to result from peripheral (cochlear) deficiencies. In contrast, *Map3k1^tm1Yxia^* mutant mice did not exhibit any observable vestibular dysfunction phenotypes, such as hyperactivity, head-tossing or circling behavior.

### *Map3k1^tm1Yxia^* mutant mice display rapid degeneration of OHCs and spiral ganglion neurons

**Fig. 5.**
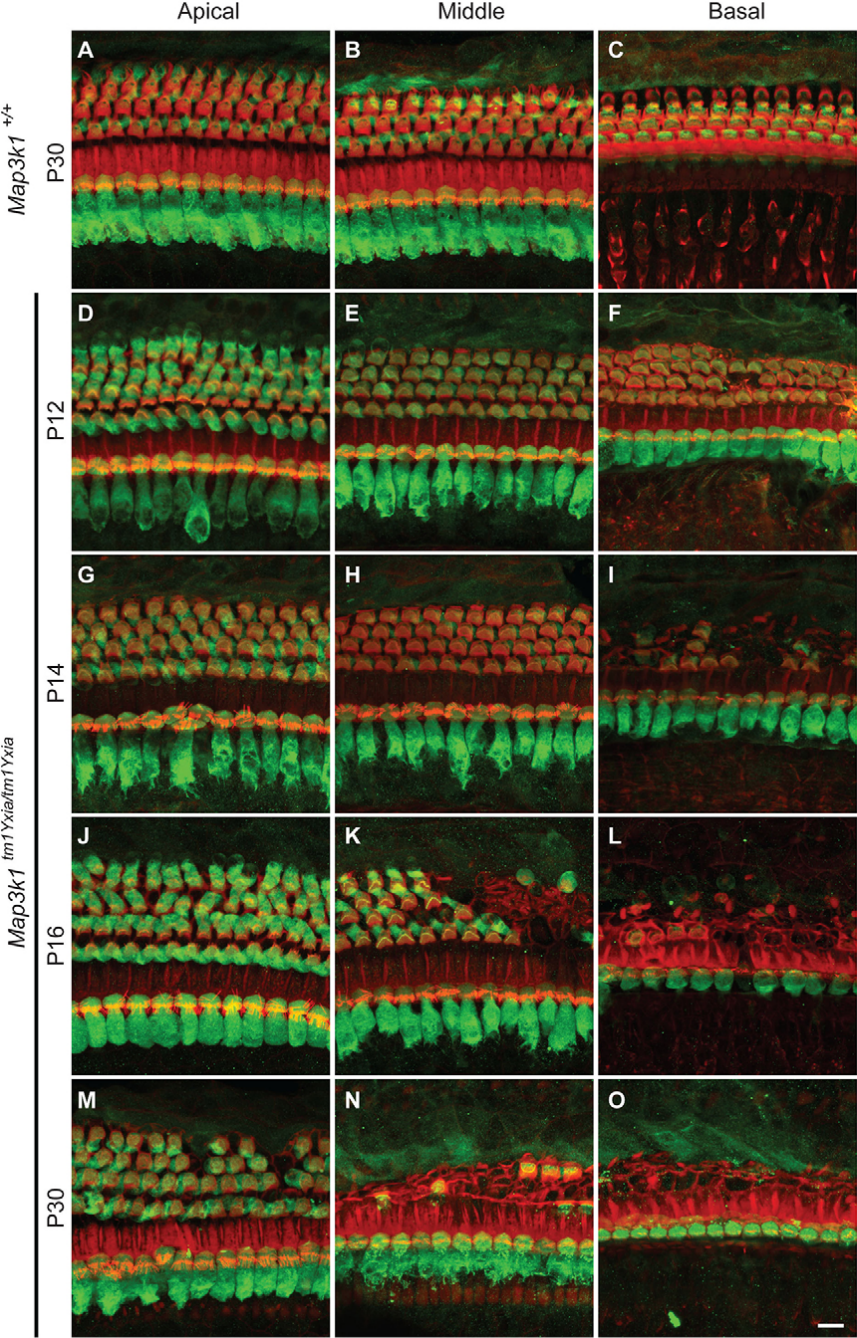
**Outer hair cells (OHCs) in *Map3k1^tm1Yxia^* mutant mice degenerate as early as P14.** Maximum intensity projections of confocal Z-stacks of whole-mount cochleae labeled with the anti-myosin-VIIa antibody (green) and phalloidin (red) are shown. (A-C) Representative images from the apical, middle and basal turns of the organ of Corti (OC) of a wild-type control mouse at P30. (D-O) Images of the OC from the three turns of the cochlea of *Map3k1^tm1Yxia^* mutant mice at P12 (D-F), P14 (G-I), P16 (J-L) and P30 (M-O). The hair cells appear to have normal development and morphology at P12 in the apical and middle cochlear turns in *Map3k1^tm1Yxia^* mutant mice. Initial signs of OHC degeneration are evident in the basal turn (F). At P14, obvious degeneration of OHCs was observed in *Map3k1^tm1Yxia^* mutant mice. (K,L) Severe OHC degeneration can be observed by P16 in the middle (K) and basal (L) turns. The OHC loss progresses rapidly and, by P30, severe degeneration is evident in all three cochlear turns. In contrast, inner hair cells (IHCs) remained intact along the length of the cochlea. Scale bar: 10 μm.

**Fig. 6.**
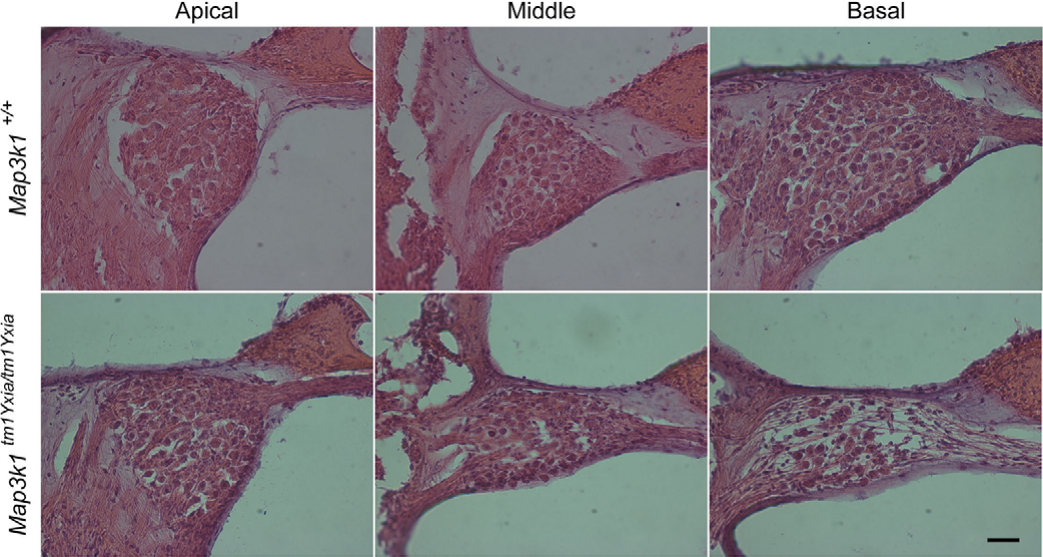
***Map3k1^tm1Yxia^* mice at P90 displayed the degeneration of spiral ganglions in the basal turn.** Cochlear sections from control and *Map3k1^tm1Yxia^* mutant mice were stained with hematoxylin and eosin. Scale bar: 20 μm.

Next, we examined the morphology of the cochlear epithelium at various developmental stages to determine the underlying cause of hearing loss observed in the *Map3k1^tm1Yxia^* mutant mice. Confocal imaging of myosin-VIIa-labeled OC of *Map3k1^tm1Yxia^* mice revealed normal development of IHCs and OHCs, which were indistinguishable from those of wild-type and heterozygous mice until P12 (Fig. 5). By P14, *Map3k1^tm1Yxia^* homozygous mice displayed obvious degeneration of OHCs in the basal turn, and by P16, no intact OHCs were observed in the basal turn (Fig. 5L). Furthermore, by P16, a varying degree of OHC degeneration was observed along the length of the cochlea (Fig. 5J-L). However, by P30, almost all of the OHCs in the basal and middle turn of the cochlea were degenerated, and many OHCs in the apical coil were also degenerated (Fig. 5M-O). In contrast, the IHCs appeared largely intact even at P75 (Fig. 5 and data not shown). The loss of OHCs in *Map3k1^tm1Yxia^* mutant mice was followed by progressive degeneration of the spiral ganglion, which was more pronounced in the basal region at P90 (Fig. 6). Thus, the elevated hearing thresholds observed in MAP3K1-deficient mice are likely to be caused by the rapid degeneration of OHCs. These results suggest that MAP3K1 function is essential for the maintenance of OHCs in the mouse auditory system.

### Genes of the FGF signaling pathway are downregulated in *Map3k1* mutant mice

**Fig. 7.**
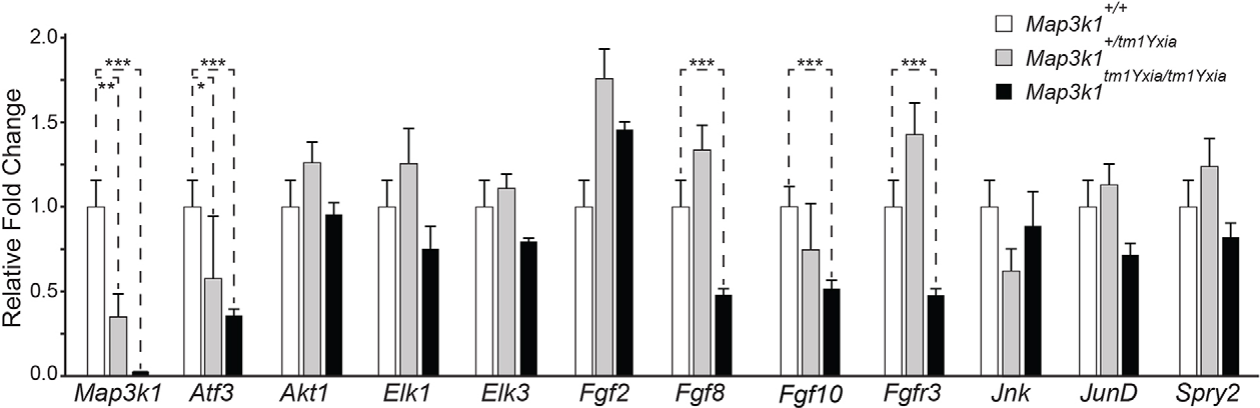
***Fgfr3*, *Fgf8*, *Fgf10* and *Atf3* genes are downregulated in the organ of Corti (OC) of *Map3k1^tm1Yxia^* mutant mice.** Semi-quantification expression analysis of various developmental and MAP3K1-mediated signaling pathway genes normalized against *Gapdh* and actin (endogenous controls) at P10. As anticipated, a dose-dependent, statistically significant reduction in the expression of *Map3k1* was observed in heterozygous and homozygous mutant mice. Furthermore, the expression of *Fgfr3*, *Fgf8*, *Fgf10* and *Atf3* was also significantly abolished (Student's *t*-test, ***P<0.001), suggesting a role of *Map3k1* in regulating their expression in the inner ear (mean±s.e.m.). **P<0.01; *P<0.05.

We reasoned that the extra row of OHCs and Deiters' cells and degeneration of hair cells in *Map3k1* mutant mice is likely to be caused by impaired intracellular signaling during the development and maturation of mouse OC. Therefore, we examined the expression of various genes associated with the *Map3k1*-mediated signaling pathway (Fig. 7 and see Fig. S3) and transcriptional targets of FGF signaling, as well as the development-related genes in *Map3k1^tm1Yxia^* mutant mice at P10 (before the onset of hearing and OHC degeneration). As expected, the heterozygous mice had reduced expression of *Map3k1*, whereas the homozygous mice had no expression (Fig. 7). Among the 42 genes analyzed, we observed significant (*P*<0.001) downregulation of *Atf3*, *Fgfr3*, *Fgf8* and *Fgf10* in the inner ear of *Map3k1* mutant mice (Fig. 7). Previous studies have shown that *Fgfr3*, *Fgf8* and *Fgf10* have a role in the otic placode induction or in otic epithelium development in mice, and their functional deficits cause defects in cochlear morphogenesis and hearing loss (Hayashi et al., 2007; Mansour et al., 2009; Pannier et al., 2009). Our results suggest that MAP3K1 has an essential role in the regulation of these key cochlear morphogenesis genes during development.

## DISCUSSION

MAP3K1 is involved in the cellular response to a wide array of stimuli, including growth factors, pro-inflammatory cytokines, cell shape disturbance, microtubule disruption and a variety of cell stress signals (Cardone et al., 1997; Deak et al., 1998; Nakagami et al., 2001; Widmann et al., 1998; Xia et al., 2000; Yujiri et al., 1998), which translate into activation of the downstream JNK-, p38- and ERK-mediated MAPK pathways (Schlesinger et al., 1998; Xu et al., 1996). The full-length MAP3K1 protein is tethered to insoluble structures such as membranes or the cytoskeleton by the actin-bundling protein α-actinin (Christerson et al., 1999), and has also been found to be associated with actin fibers entering focal adhesions, so is implicated in controlling their turnover (Cuevas et al., 2003). MAP3K1 interacts directly with the signaling molecule MEK1 (Karandikar et al., 2000), which, in the mouse oocyte, has been associated with the control of microtubule organization and colocalizes with the centrosomal protein γ-tubulin (Yu et al., 2007). Interestingly, in *Map3k1^tm1Yxia^* mice, the MAP3K1–β-galactosidase fusion protein in the supporting cells of the OC also colocalized with the centrosomal protein and, therefore, might also participate in microtubule organization in the inner ear non-sensory cells and in PCP (Jones et al., 2008). There are many examples of PCP proteins, such as Vangl2, that are not expressed in the inner ear sensory hair cells, but still severely affect the stereocilia bundle orientation (Montcouquiol et al., 2006). However, in the *Map3k1^tm1Yxia^* mutant mice we did not observe any significant stereocilia bundle orientation deficit, which might reflect that *Map3k1* has either no direct role in PCP, or that there is functional redundancy with other family members.

In a parallel study, a splice-site mutation in the *Map3k1* gene in *goya* mutant mice was identified as part of an ENU mutagenesis screening program (Parker et al., 2015). *goya* mice also exhibit a progressive hearing-loss phenotype along with the supernumerary OHCs. Interestingly, when maintained on the same genetic background (C3H), the *goya* and *Map3k1^tm1Yxia^* mutant mice were found to have a similar progressive hearing-loss phenotype and were profoundly deaf by 9 weeks (Parker et al., 2015). Modulation of the phenotype of a given allele by the genetic background of an inbred strain is a well-documented phenomenon (Doetschman, 2009; Montagutelli, 2000). Intriguingly, in addition to the increased hearing loss that we observed, a study involving *Map3k1* mice on the C57BL/6J background has also shown a drastic decrease in the number of animals surviving to maturity (Warr et al., 2011), which further supports the notion of a *Map3k1* modifier gene.

Although the expression of MAP3K1 was observed in the supporting cells of the OC, *Map3k1^tm1Yxia^* mutant mice exhibited four rows of OHCs. During development, extra OHCs can be found in wild-type mice, but they are generally restricted in the apical turns of the cochlea, and their frequency can vary depending on different inbred mouse strains (Lim and Anniko, 1985), whereas, in *Map3k1^tm1Yxia^* mutant mice, the fourth row of OHCs was found throughout development and along the length of the cochlea. One possibility to explain such a phenotype includes the lack of apoptosis of a population of cells in the prosensory domain that would otherwise have undergone degeneration during development. Normally, full-length MAP3K1 acts as an anti-apoptotic protein but, when cleaved by caspase 3, becomes pro-apoptotic (Schlesinger et al., 2002). Relocalization of the MAP3K1 C-terminal 91-kDa fragment containing the kinase domain is necessary for its pro-apoptotic function; however, in the case of *Map3k1^tm1Yxia^* mice, the kinase domain is replaced with the *lacZ* domain, rendering it incapable of inducing apoptosis in the supporting cells of the developing OC.

Alternatively, the supernumerary OHCs in *Map3k1^tm1Yxia^* could stem from the deficit in the control of the prosensory domain size during embryogenesis. The sensory epithelial cell differentiation initiates with specification of the prosensory domain in the otocyst. Many signaling molecules, including the sonic hedgehog, Notch and Wnt pathway genes, act in concert to form a highly specialized patterned sensory epithelium. Canonical Wnt (Wnt/β-catenin) signaling is also essential for the specification of the otic placode because either conditional deletion or activation of β-catenin in Pax2-positive ectodermal cells or Foxg1-positive placodal cells results in a substantial reduction or expansion of the size of the otic placode, respectively (Freyer and Morrow, 2010; Ohyama et al., 2006). MAP3K1, via direct interaction with Axin1, is known to modulate Wnt/β-catenin signaling pathway activity (Sue Ng et al., 2010). Previously, fibroblasts from *Map3k1^tm1Yxia^* mutant mice displayed a tenfold increase in transcription factor *Lef/Tcf* reporter activity in response to *Wnt3a* expression compared to an only threefold increase observed in wild-type cells (Jin et al., 2013). Because Wnt expression regulates proliferation in the early prosensory domain and hair cell differentiation in the later stage, the level of Wnt/β-catenin signaling can affect hair cell formation in a dose-dependent manner (Jacques et al., 2012). It is plausible that, during development, Wnt/β-catenin signaling might have resulted in relative overactivation of downstream transcription factors, leading to the expansion of the prosensory domain and excessive differentiation of OHCs in *Map3k1^tm1Yxia^* mutant mice. Future work using cell-specific markers and cochlear tissue from various embryonic developmental stages will provide knowledge regarding the role of MAP3K1 in sensory-cell specification and differentiation.

*Map3k1^tm1Yxia^* mutant mice exhibit downregulation of at least four genes, which are known for their role in inner-ear development and function. ATF3, a transcription factor that is induced in response to a number of stress stimuli (Hai et al., 1999; Kyriakis et al., 1994; Liang et al., 1996), is significantly downregulated in *Map3k1^tm1Yxia^* mutant mice. ATF3 is also implicated in the survival, repair and neurite outgrowth in association with heat shock protein 27, Akt and Jun (Nakagomi et al., 2003; Pearson et al., 2003). Also, *Fgfr3* mutant cochlea show neuronal wiring pattern disruption (Puligilla et al., 2007). However, in *Map3k1^tm1Yxia^* mutant mice, we did not observe any gross deficit in the neuronal wiring. Furthermore, the DPOAE data are suggestive of a functional deficit of hair cells in *Map3k1^tm1Yxia^* mutant mice. These results suggest that downregulation of *Atf3* is unlikely to be the reason for the supernumerary OHCs and Deiters' cells, and hearing deficits, observed in *Map3k1^tm1Yxia^* mutant mice.

Before the onset of hair-cell degeneration at P10, we found significant downregulation of *Fgfr3*, *Fgf8* and *Fgf10* expression in the *Map3k1^tm1Yxia^* mutant mouse OC cells. Dysfunction of *Fgfr3* in mice, either due to loss (Hayashi et al., 2007) or gain (Mansour et al., 2009; Pannier et al., 2009) of function, results in hearing loss. Interestingly, both *Fgfr3* mutant alleles have supernumerary OHCs (Hayashi et al., 2007; Mansour et al., 2009), as is observed in *Map3k1^tm1Yxia^* mutant mice. This implies that, in inner-ear development, *Map3k1* and *Fgfr3* might participate in the same signaling cascade to control the precise cytoarchitecture of the OC. However, in contrast to *Fgfr3* mutant mice, which have either loss of pillar or Deiters' supporting cells in the OC (Hayashi et al., 2007; Mansour et al., 2009; Pannier et al., 2009), *Map3k1^tm1Yxia^* mutant mice do not exhibit any obvious deficit in the differentiation of pillar cells; rather, they have an extra row of Deiters' cells. The phenotypes of overproduction of OHCs and loss of pillar cells reported for the gain-of-function allele of *Fgfr3* phenotype is remarkably similar to *Sprouty2*, an antagonist of FGF signaling, mutant mice (Shim et al., 2005). Intriguingly, the hearing-loss phenotype in *Sprouty2* mutant mice can be partially rescued by genetically reducing the *Fgf8* expression level (Shim et al., 2005). Furthermore, reduction of *Fgf10* expression reverted the fate-switched supporting cells back and restored hearing in *Fgfr3* mutant mice (Mansour et al., 2013). Besides *Fgfr3*, *Fgf8* and *Fgf10* are also downregulated in *Map3k1^tm1Yxia^* mutant mice, which could account for the grossly intact supporting cells in these mutant mice as compared to *Fgfr3* mutant mice.

Our studies further highlight the complexity of the signaling pathway(s) required for formation of the precise cytoarchitecture of mouse OC and the maintenance of OHCs. We also found that MAP3K1 function was necessary for regulation of the FGF-mediated pathway in the auditory system and hearing in mice.

## MATERIALS AND METHODS

### *Map3k1* mutant mice

*Map3k1^tm1Yxia^* mice were generated as described previously (Xia et al., 2000; Zhang et al., 2003). In summary, the targeting vector replaced the entire kinase domain in the *Map3k1* locus, resulting in the formation of a MAP3K1–β-galactosidase fusion protein. These targeted embryonic stem (ES) cells (*Map3k1^+/tm1Yxia^*) were injected into mouse blastocysts, and the resulting chimeras were crossed with C57BL/6 mice to obtain mice with germline transmission of the *Map3k1^tm1Yxia^* allele. All experiments were approved by the Animal Care and Use Committees at the University of Maryland, School of Medicine in accordance with the National Institutes of Health (NIH) Guide for the Care and Use of Laboratory Animals.

### ABR and DPOAE measurements

Hearing function was evaluated by ABR analyses at two different time points (P16 and P30) in mice of all the three genotypes, i.e. wild-type, heterozygous and *Map3k1^tm1Yxia^* homozygous mice. Mice were anesthetized with intraperitoneal injections of Avertin (0.4-0.75 mg/g body weight, Sigma-Aldrich, St Louis, MO). All recordings were performed in a sound-attenuated chamber using an auditory-evoked potential diagnostic system RZ6 (Tucker-Davis Technologies Inc., Alachua, FL) as previously described (Nayak et al., 2013). Experiments represent the mean and standard error of mean (s.e.m.) of three or more animals. Significance was analyzed using Student's *t*-test.

DPOAE recordings were performed at P30 with an acoustic probe (ER-10C, Etymotic Research, Elk Grove Village, IL) using a DP2000 DPOAE measurement system version 3.0 (Starkey Laboratory, Eden Prairie, MN). Two primary tones, with a frequency ratio of f2/f1=1.2, where f1 represents the first tone and f2 represents the second, were presented at intensity levels L1=65 dB sound pressure level (SPL) and L2=55 dB SPL. Also, f2 was varied in one-eighth octave steps from 8 to 16 kHz. DP grams comprised 2f1–f2 DPOAE amplitudes as a function of f2.

### Confocal imaging

The temporal bones from the control and mutant mice were isolated and fixed for 1 h at room temperature or overnight at 4°C in 4% paraformaldehyde (Electron Microscopy Sciences, Hatfield, PA), followed by three washes with phosphate buffered saline (PBS). Finely dissected cochlear coils were permeabilized and blocked in 5% BSA, 2% normal goat serum and 0.1% Triton X-100 in PBS for 1 h. The tissue samples were washed and probed with primary antibody overnight at 4°C. We used the following primary antibodies in our studies: β-galactosidase (1:1000 dilution; MP Biomedicals, Solon, OH), myosin VIIa (1:200; Proteus BioSciences, Ramona, CA), synaptophysin (1:200; Abcam, Cambridge, MA), NF-200 (1:200; Sigma-Aldrich), Prox1 (1:100; Millipore, Billerica, MA) and Pericentrin (1:200; Millipore, Billerica, MA). After three washes with PBS, samples were probed with a fluorescently labeled Alexa-Fluor-488 or -546 secondary antibody (1:500; Life Technologies, Grand Island, NY) for 1 h at room temperature. Rhodamine-phalloidin or Alexa-Fluor-647-conjugated phalloidin (1:250; Life Technologies, Grand Island, NY) was used to label actin. Samples were mounted using ProLongGold (Life Technologies, Grand Island, NY) and viewed under an LSM 700 confocal microscope (Zeiss Microimaging Inc., Thornwood, NY) using a ×63, 1.4 N.A. oil-immersion lens.

### Scanning electron microscopy (SEM)

For SEM studies, the inner ears were isolated at P14 and fixed for 1.5 h in a fixative containing 2.5% glutaraldehyde and 2 mM CaCl_2_ in 0.1 M sodium cacodylate buffer, and later washed three times with 0.1 M sodium cacodylate buffer. The inner ears were post-fixed in 1% osmium tetroxide in 0.1 M sodium cacodylate buffer for 1 h at room temperature. After washing three times with PBS buffer, inner ears were decalcified by incubating in 0.25 M EDTA for 1-2 days at 4°C. The samples were then finely dissected to expose the sensory epithelium and to remove the tectorial membrane and the stria vascularis. The cochlear tissues were then dehydrated in gradient alcohol changes, critical point dried, sputter coated with platinum, and imaged on a field-emission SEM.

### Cochlear explants and FM1-43 dye uptake

OC explants from P3 wild-type and *Map3k1^tm1Yxia^* mutant mice were finely dissected and cultured on collagen-coated glass-bottom Petri dishes (MatTek Corporation, Ashland, MA) in DMEM medium supplemented with 10% fetal bovine serum (Life Technologies, Grand Island, NY) and 10 mg/ml ampicillin (Millipore, Billerica, MA) at 37°C and 5% CO_2_. The explants were kept *in vitro* for 2 days to allow for complete adhesion to the dish. To test the FM1-43 dye {N-(3-triethylammoniumpropyl)-4-[4-(dibutylamino)-styryl]pyridinium dibromide} (Life Technologies, Grand Island, NY) uptake by the hair cells, the culture was exposed to 3.0 µM FM1-43 in Hank's Balanced Salt solution (HBSS) for 15 s and then quickly washed three times with HBSS. The culture was mounted with Fluoro-Gel (Electron Microscopy Sciences, Crofton, MD) and immediately imaged under an LSM 700 confocal microscope (Zeiss Microimaging Inc.).

### Cryosections

Hemisected heads of P0 mice or inner ears of P10 and older mice were collected and fixed in 4% paraformaldehyde (PFA) at 4°C overnight. For older mice, the temporal bones were decalcified in 0.25 M EDTA for 1 to 2 days at 4°C. The inner ears were then equilibrated with 30% sucrose in PBS overnight at 4°C, embedded in OCT, and immediately frozen by placing the block on an ethanol/dry-ice mix. The frozen tissue blocks were sectioned with a cryostat at 14 μm thickness.

### Reverse transcriptase PCR (RT-PCR) and real-time PCR

Total RNA was isolated from fine dissected OC isolated from P10 inner ear tissue of wild-type, heterozygous and *Map3k1^tm1Yxia^* homozygous mice (five mice each) using the RiboPure RNA isolation kit (Life Technologies, Grand Island, NY) and cDNA was prepared using an oligo-dT primer and SMARTScribe Reverse Transcriptase enzymes (Clontech, Mountain View, CA). To determine the differential expression of various genes, SYBR-Green-based real-time primers (available upon request) were designed using Integrated DNA Technologies online PrimeTime qPCR assay design tool (http://www.idtdna.com/Scitools/Applications/RealTimePCR/). The real-time PCR assays were performed in triplicate using an ABI StepOnePlus Real-Time thermalcycler (Life Technologies, Grand Island, NY). C_T_ values were normalized using *Gapdh* and actin as an endogenous control, and fold changes of different genes were calculated using SABiosciences online software (http://pcrdataanalysis.sabiosciences.com/pcr/arrayanalysis.php). The genes with a twofold change and with a *P*-value less than 0.05 based on a Student's *t*-test analysis were considered significant.

### X-gal staining

The inner ears were isolated from wild-type and *Map3k1^tm1Yxia^* heterozygous mice at P30 and were fixed for 10 min at room temperature in LacZ fixative (1% formaldehyde, 0.2% glutaraldehyde and 0.02% NP-40 in PBS). Then, the samples were washed twice with PBS containing 0.02% NP-40 and 2 mM MgCl_2_, 10 min each, followed by incubation at 37°C overnight in a staining solution [1 mg/ml X-Gal, 5 mM K_3_Fe(CN)_6_, 5 mM K_4_Fe(CN)_6_ and 0.1 M MgCl_2_]. Following staining, samples were washed in PBS, decalcified and cryosectioned as described above. The sections were counterstained with hematoxylin and eosin and imaged using a ×40 oil immersion lens on a Zeiss Axioplan Apotome-equipped microscope.
